# Continuous Monochromatic Blue Light Exacerbates High-Fat Diet-Induced Kidney Injury via Corticosterone-Mediated Oxidative Stress

**DOI:** 10.3390/antiox12051018

**Published:** 2023-04-28

**Authors:** Wenji Ren, Zixu Wang, Jing Cao, Yulan Dong, Tuanjie Wang, Yaoxing Chen

**Affiliations:** 1 Department of Animal Anatomy and Histoembryology, College of Veterinary Medicine, China Agricultural University, Beijing 100193, China; 2 China Institute of Veterinary Drug Control, Beijing 100081, China

**Keywords:** monochromatic light, high-fat diet, kidney, oxidative stress, inflammation, corticosterone

## Abstract

Excessive illumination is one of the most severe environmental factors that impacts the organism. There is growing evidence that obesity significantly contributes to the onset of chronic kidney disease. However, the effect of continuous light on the kidney and which color can produce an apparent phenomenon remains elusive. In this study, C57BL/6 mice given either a normal diet (LD-WN) or a high-fat diet (LD-WF) were subjected to a light cycle of 12 h of illumination followed by 12 h of darkness for 12 weeks. Meanwhile, 48 high-fat diet mice were given a 24 h monochromatic light exposure of varying colors (white, LL-WF; blue, LL-BF; green, LL-GF) for 12 weeks. As expected, the LD-WF mice showed significant obesity, kidney injury, and renal dysfunction compared with the LD-WN group. LL-BF mice had worse kidney injury than LD-WF mice, including higher *Kim-1* and *Lcn2*. The kidney of the LL-BF group showed marked glomerular and tubular injury, with decreased levels of *Nephrin*, *Podocin*, *Cd2ap*, and *α-Actinin-4* compared to LD-WF. LL-BF also reduced the antioxidant capacity, including GSH-Px, CAT, and T-AOC, increased the production of MDA, and inhibited the activation of the NRF2/HO-1 signaling pathway. Furthermore, LL-BF upregulated the mRNA levels of the pro-inflammatory factors *Tnf-α*, *Il-6*, and *Mcp-1*, decreasing the inhibitory inflammatory *Il-4* expression. We observed increased plasma corticosterone (CORT), renal glucocorticoid receptors (GR) expression, *Hsp90*, *Hsp70*, and *P23* mRNA levels. These findings suggested that LL-BF increased CORT secretion and affected glucocorticoid receptors (GR) in comparison to the LD-WF group. Moreover, in vitro research demonstrated that CORT treatment increased oxidative stress and inflammation, which was counteracted by adding a GR inhibitor. Thus, the sustained blue light worsened kidney damage, possibly by inducing elevated CORT and increasing oxidative stress and inflammation via GR.

## 1. Introduction

Obesity caused by a high-fat diet is one of the major health risks worldwide [1]. Patients with central obesity are prone to metabolic syndrome (MetS), mainly characterized by hyperglycemia, hyperlipidemia, and hypertension, and the probability of cardiovascular disease-related death is higher than before [2,3]. Obesity has recently emerged as a significant risk factor for diabetes, which frequently results in chronic kidney disease (CKD) and even causes end-stage renal disease (ESRD) [4,5]. Although much progress has been made in our standing and proper treatments of CKD and diabetes, it remains a potential clinical issue.

The increase in disease and mortality due to environmental pollution, including light pollution, has become a global crisis, especially in developing countries. Optical information affects the normal operation of almost all biological systems [6], as light does more than aid in visual acuity. It also provides a non-visual physiological impetus. Thus, organisms coordinate physiological and behavioral changes by synchronizing their intrinsic sleep patterns with 24 h light changes [7]. Abnormal light exposure can have adverse effects on individuals. Research has shown that excessive illumination of various intensities and wavelengths can cause asynchrony of circadian rhythms [8]. The kidney is an organ that is closely associated with circadian rhythms. In humans, exposure to excessive light can induce essential hypertension in the mother’s male heirs [9]. Further, the development of hypertension is closely related to the kidney [10]. However, there is a paucity of studies on the effects of continuous light exposure on the kidney.

In mammals, prolonged exposure to light affects circadian rhythms [11], sleep–wake cycles, and hormone secretion (such as melatonin and corticosterone (CORT)) [12]. It has been shown that exposure to continuous bright light can reduce tyrosine hydroxylase (TH)-positive dopamine neurons and thus influence the prevalence of Parkinson’s disease [13]. Moreover, it should be noted that the function of spectrum distribution in physiological regulation has been confirmed [14]. Our previous study showed that low-intensity blue light at night caused inflammation and oxidative stress in the hippocampus, which manifested as memory impairment in mice [15]. Furthermore, we reported that continuous blue light exposure induced hepatic steatosis in HFD-treated mice [16]. Therefore, it is necessary to investigate the effect of ambient light on renal injury.

In recent studies, excessive lipid accumulation can exacerbate kidney damage by causing inflammation, oxidative stress, and mitochondrial dysfunction [17]. Information is projected to the suprachiasmatic nucleus (SCN) in the hypothalamus when the retina receives light signals. The SCN is the mammalian brain’s circadian pacemaker, which controls rhythmic changes in hormone secretion (melatonin and glucocorticoids) and physiology functions of peripheral organs [18]. The kidney is controlled by circadian rhythms, and its certain functions exhibit distinct rhythmic changes [19,20]. Interestingly, recent research revealed that the kidney is second only to the liver in the expression of circadian genes [21]. Thus, there may be some potential effects of abnormal light exposure on the kidneys.

Therefore, the effects of continuous exposure to different wavelengths of light on the kidney remain to be determined. This study sought to explore the effects of long-term exposure to monochromatic light on kidney damage in obese mice and its probable pathways, thus developing a novel theoretical framework for the relationship between light pollution and the kidney.

## 2. Materials and Methods

### 2.1. Animals and Light Patterns

All animal studies were handled following the Animal Welfare Guidelines issued by the Chinese Agricultural University Agricultural Research Institute (Approval No. AW18079102-2). Under conventional conditions (temperature at 23 ± 1 °C, 50–60% relative humidity in the air), three-week-old male C57BL/6 mice (Charles River Co., Ltd. Beijing, China) were housed with a 12 h light followed by a 12 h dark alternating cycle (lights on at 8:00 a.m.). Each plastic cage held three mice.

In a week after acclimatizing, a total of the following five groups of mice were selected at random using different types of illumination (white, blue, green light): (1) Normal low-fat diet (derives 10% calories from fat, Charles River Co., Ltd. Beijing, China) with conventional light-dark cycle of white light (LD-WN; 450–465 nm; *n* = 6). (2) High-fat diet (HFD, derives 60% calories from fat, Hfk Bioscience Co., Ltd., Beijing, China) with LD cycle of white light (LD-WF; *n* = 12); (3) HFD with continuous 24 h white light (LL-WF; *n* = 12); (4) HFD with continuous 24 h monochromatic blue light (LL-BF, peak at 450 nm; *n* = 12); (5) HFD with continuous 24 h monochromatic green light (LL-GF, peak at 517 nm; *n* = 12). Lights were implemented using LEDs (12V-5050-60D, Junsheng Lighting Technology Co., Ltd. Zhongshan, China), and the intensity of all types of light is 150 lux. During dietary and light treatment, body weight, food, and water intake were tracked weekly for 12 weeks during dietary and light treatment. Kidneys from all groups were harvested immediately after euthanasia for histologic and biochemical analysis at the end of the 12-week experiments.

### 2.2. Blood Biochemical Analysis

Mice were fasted for 12 h before blood samples were taken in the morning. Serum creatine (Scr) was quantified using a robotic biochemical analysis tool (Mindray Medical Inc., Shenzhen, China). Blood glucose was measured using test strips (Yuwell Medical Equipment and Supply Co., Ltd. Suzhou, China). Each group included six samples, which were analyzed three times.

### 2.3. Urine Examination

The concentrations of urinary albumin, urinary creatine (Ucr), and uric acid (UA) were determined separately using commercially available assay kits (C035-2-1; C011-2-1; C012-2-1, Nanjing Jiancheng Bioengineering Institute, Nanjing, China). The level of urine albumin to creatinine ratio (UACR) was normalized. All tests were conducted according to the instructions provided by the manufacturers.

### 2.4. Histological Analysis

Once kidney segments were harvested, they were immediately fixed in 4% paraformaldehyde in 0.1 mol/L phosphate-buffered saline (pH = 7.4, 4 °C) for 48 h, embedded in paraffin, and sectioned with a 5 μm thickness (RM2235, Leica, Germany). Hematoxylin-eosin (H&E; G1120, Solarbio Life Sciences, Beijing, China) staining and Periodic acid-Schiff (PAS; G1281, Solarbio Life Sciences, Beijing, China) staining were performed to evaluate kidney injury and degree of renal steatosis. Sirius Red staining (G1472, Solarbio Life Sciences, Beijing, China) was performed to estimate renal fibrosis. In the kidney, at least 30 randomly picked fields in five separate kidney samples were photographed at 400× magnification with a scale of 25 μm using a microscope (BX51, Olympus, Tokyo, Japan). An analysis of the data was carried out using Image-Pro Plus (Media Cybernetics, Rockville, MD, USA).

### 2.5. Immunohistochemistry (IHC) Staining and Immunofluorescence (IF)

Samples were heated to 98 °C in 0.1 M citrate buffer (pH 6.0) for 10 min when the antigens were recovered. Endogenous peroxidase was blocked with 10% H_2_O_2_ for 20 min, and nonspecific antigens were blocked with goat serum (SL038, Solarbio Life Sciences, Beijing, China) for 30 min at 37 °C. Kidney paraffin sections were incubated in mouse anti-glucocorticoid receptor antibody (GR, 1:300, 66904-1-Ig, Proteintech, Chicago, IL, USA), rabbit anti-proliferating cell nuclear antigen (PCNA, 1:300, 10205-2-AP, Proteintech; Chicago, IL, USA) primary antibody overnight at 4 °C. The sections were then rinsed in 0.01 M PBST (pH 7.4), and slides were then incubated with biotin-conjugated secondary antibodies (1:300, Beyotime, Shanghai, China) for 2 h at room temperature. After washing, the tissues were incubated with streptavidin-horseradish peroxidase (1:300, Sigma, St. Louis, MO, USA) for 1.5 h at room temperature. Coloration was performed using 0.01 M PBST containing 0.05% 3′, 3-diaminobenzidine tetrahydrochloride (DAB; Sigma), and 0.003% H_2_O_2_ for 10 min in the dark for several seconds. Immunohistochemical-positive cells were brown, and nuclei were stained with hematoxylin.

For immunofluorescence analysis, the primary antibody was rabbit anti-α-SMA (1:150, 14395-1-AP, Proteintech). The secondary antibody was consistent with immunohistochemistry. Afterward, the sections were incubated with an Atto-594-streptavidin (1:100, 68606, Sigma) for 1 h at room temperature. Nuclei were counterstained with DAPI (D9542, Sigma). Representative images were captured with an OLYMPUS microscope. Data were analyzed using Image-Pro Plus software (Media Cybernetics, Rockville, MD, USA).

### 2.6. RNA Isolation and Quantitative Real-Time (RT)-PCR

Total RNA was extracted from kidney tissues (*n* = 6) using a Trizol reagent according to the manufacturer’s instructions (CW0580, CoWin Biotech Co., Inc., Beijing, China). Then, 1000 ng of total RNA was reverse-transcribed in a final 20 μL with HiScript Ⅱ First-Strand cDNA Synthesis Kit (R212-01, Vazyme Biotech Co., Ltd., Nanjing, China). The reverse transcription reaction was processed at 50 °C for 15 min and 85 °C for 2 min. qRT-PCR amplification was performed using AceQ qPCR SYBR Green Master Mix (Q111-02, Vazyme Biotech Co., Ltd., Nanjing, China) on StepOne Real-time PCR systems (ABI, Boston, MA, USA) in triplicate, and non-template controls were run under the same conditions for each assay. The PCR reaction was carried out with an initial denaturation step of 95 °C for 5 min, followed by 40 cycles of 95 °C for 10 s and 60 °C for 30 s. Relative mRNA expression levels were calculated using the 2^—ΔΔCt^ method and normalized to *Gapdh* expression levels. The primer sequences used are shown in Table 1.

### 2.7. Western Blotting

Total proteins were extracted from mice kidney tissues using RIPA buffer (P0013B, Beyotime, Shanghai, China). Equal amounts of protein (30 μM) were separated by sodium dodecyl sulfate-polyacrylamide gel electrophoresis (SDS-PAGE). Samples were transferred to the PVDF membrane and blocked with a blocking buffer (5% nonfat powdered milk in TBST) for 1.5 h. After blocking, the membranes were incubated with the following primary antibodies: α-smooth-muscle actin-α (α-SMA) rabbit monoclonal Ab (1:2000, Proteintech), iNOS rabbit monoclonal Ab (1:1000, CST), nuclear factor erythroid (NRF2) rabbit monoclonal Ab (1:1000, Proteintech), HO-1 rabbit monoclonal Ab (1:1000, CST), TLR4 mouse monoclonal Ab (1:1000, Santa Cruz), total and phosphorylated-P65 rabbit monoclonal Ab (1:1000, CST), Glucocorticoid receptor (GR) mouse monoclonal Ab (1:20,000, Proteintech), B cell lymphoma-2 (BCL2) mouse monoclonal Ab (1:3000, Proteintech), BCL2-associated X (BAX) mouse monoclonal Ab (1:8000, Proteintech), Cleaved Caspase-3 mouse monoclonal Ab (1:1000; CST). Mouse monoclonal anti-β-actin Ab (1:8000, CWBIO, Beijing, China) was used as the loading control. The membranes were incubated with the primary antibodies overnight at 4 °C. After washing in TBST (pH 7.4), they were incubated with horseradish peroxidase-conjugated goat anti-rabbit or mouse IgG (1:8000, CWBIO, Beijing, China) for 2 h at room temperature. The protein bands were detected using an enhanced chemiluminescence kit (WBKLS0100. Millipore, Billerica, MA, USA). Images were captured using the ChemiDoc MP imaging system (Bio-rad). Each sample was assayed in three independent experiments.

### 2.8. Measurements of Antioxidant Activity and Lipid Peroxidation

Clarified kidney lysate (*n* = 6) was obtained by rapidly homogenizing and centrifuging at 4 °C (12,000 r for 5 min) and stored in aliquots at −80 °C. The activities of total antioxidant capacity (T-AOC), Glutathione peroxidase (GSH-Px), catalase (CAT), and superoxide dismutase (SOD), and the level of malondialdehyde (MDA) were measured using commercial kits (Beyotime Biotechnology Co., Ltd. Shanghai, China) following the manufacturer’s instructions.

### 2.9. Enzyme-linked Immunosorbent Assay

The plasma corticosterone (CORT) levels were determined using a competitive ELISA kit (Uscn Life Science, Inc., Wuhan, China). Absorbance was read at 450 nm with a microplate reader (Infinite M200 Pro, TECAN, Mannedorf, Switzerland). All treatments were conducted at least three times, and the CV values within and between groups should be less than 9% and 11%.

### 2.10. Cell Culture

Human renal proximal tubular cell line (HK-2) cells (CRL-2190, ATCC, Rockville, MD, USA) were cultivated according to culture method recommendations. HK-2 cells were exposed to DMEM (containing 4.5 g/L D-glucose) supplemented with 10% fetal bovine serum (FBS, Gibco) and 1% (*v*/*v*) penicillin-streptomycin. All cells were maintained at a 37 °C incubator with 5% CO_2_. Cells were treated with 10 μM corticosterone (CORT, Sigma-Aldrich, St. Louis, MO, USA). To explore the intracellular signaling pathway activated by CORT, we added 10 μM RU486 (an inhibitor of glucocorticoid receptors, MCE, Weehawken, NJ, USA) into HK-2 cells before the addition of exogenous CORT. In total, 24 h later, cells were collected for Western blot assay. HK-2 cells were plated at a density of 2 × 10^5^ cells/well in 6-well plates for protein expression studies.

### 2.11. Statistical Analyses

Mice were distributed to each group randomly for all mouse investigations. Blinded scoring of immunostaining and histology was carried out. The data were shown as the mean ± standard error of the mean (SEM) and first analyzed using SPSS version 25.0 (SPSS, Inc., Chicago, IL, USA) for normal distribution. The statistically significant difference was assessed using unpaired Student’s *t*-tests (comparison of two groups) or one-way ANOVA (comparison of three or more groups). Statistical tests were conducted using GraphPad Prism software version 8.0 (GraphPad, La Jolla, CA, USA).

## 3. Results

### 3.1. Renal Function Is Significantly Worse in HFD Mice under Continuous Monochromatic Blue Light Exposure

**Figure 1:**
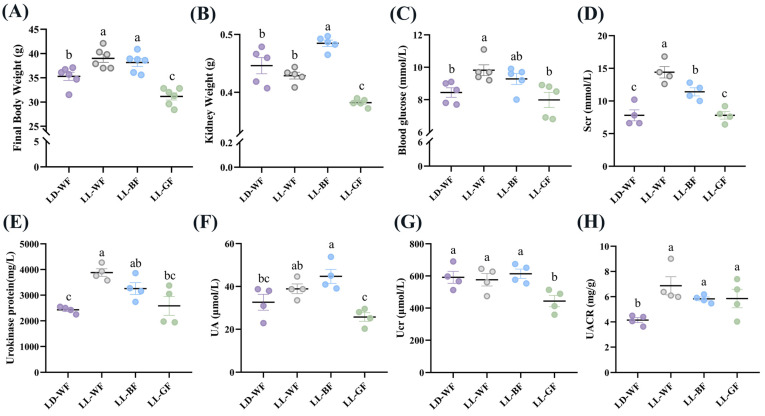
Effects of continuous monochromatic light exposure on renal function. (**A**) Body weight; (**B**) kidney weight; (**C**) fasting blood glucose changes (*n* = 5) at the end of 12 weeks of high-fat diet feeding under different wavelengths of continuous monochromatic light exposure; (**D**) Scr; (**E**) Urokinase protein; (**F**) UA; (**G**) Ucr; (**H**) UACR (*n* = 4) in these mice. LD-WF: high-fat diet mice under light-dark cycle with white light; LL-WF: high-fat diet mice under continuous white light; LL-BF: high-fat diet mice under continuous monochromatic blue light; LL-GF: high-fat diet mice under continuous monochromatic green light. These results are shown as means ± SEM. Values without the same letter were significantly different (*p* < 0.05) from each other.

To investigate the effects of continuous light exposure on renal function, male C57BL/6 mice were fed with HFD or not. We also raised them under light-dark cycles and continuous white, blue, and green light exposure for 12 weeks. As shown in Figure S1, the LD-WF mice showed a higher rate of final body weight by 25.5% (Figure S1A, *p* < 0.001) and kidney weight (Figure S1B, *p* < 0.01). Moreover, the blood glucose levels (Figure S1C, *p* < 0.05) in the LD-WF mice and serum creatinine (Scr, Figure S1D, *p* < 0.05) concentrations increased noticeably and significantly. The same trend was shown in urokinase protein excretion (Figure S1E, *p* < 0.001), uric acid (UA, Figure S1F, *p* < 0.01), urinary creatinine (Ucr, Figure S1G, *p* < 0.05) and the urinary albumin to creatinine ratio (UACR, Figure S1H, *p* < 0.05). All these are signs of renal dysfunction in the LD-WF group. Since the LD-WF mice caused renal dysfunction, we next examined the changes in different wavelengths of light exposure. The results showed that continuous blue light raised a more significant rate of final body weight (Figure 1A, *p* < 0.05) and kidney weight (Figure 1B, *p* < 0.01) than LD-WF. For the blood and urine profiles, white light and blue light aggravated the level of feeding-induced, Scr (Figure 1D, *p* < 0.001 in LL-WF, *p* < 0.01 in LL-BF), urokinase protein (Figure 1E, *p* < 0.001 in LL-WF, *p* < 0.05 in LL-BF), and UACR (Figure 1H, *p* < 0.01 in LL-WF, *p* < 0.05 in LL-BF) concentrations compared with LD-WF mice. However, the blood glucose level was only elevated in LL-WF (Figure 1C, *p* < 0.05), not LL-BF. UA levels increased only in LL-BF (Figure 1F, *p* < 0.05), not in white light. Ucr did not show significant changes between white and blue light (Figure 1G, *p* > 0.05). Taken together, LL-BF exerted detrimental effects on renal functions, which facilitate HFD-induced renal dysfunctions.

### 3.2. Continuous Monochromatic Blue Light Aggravated Renal Histological Injury and Apoptosis in the LD-WF Mice

**Figure 2:**
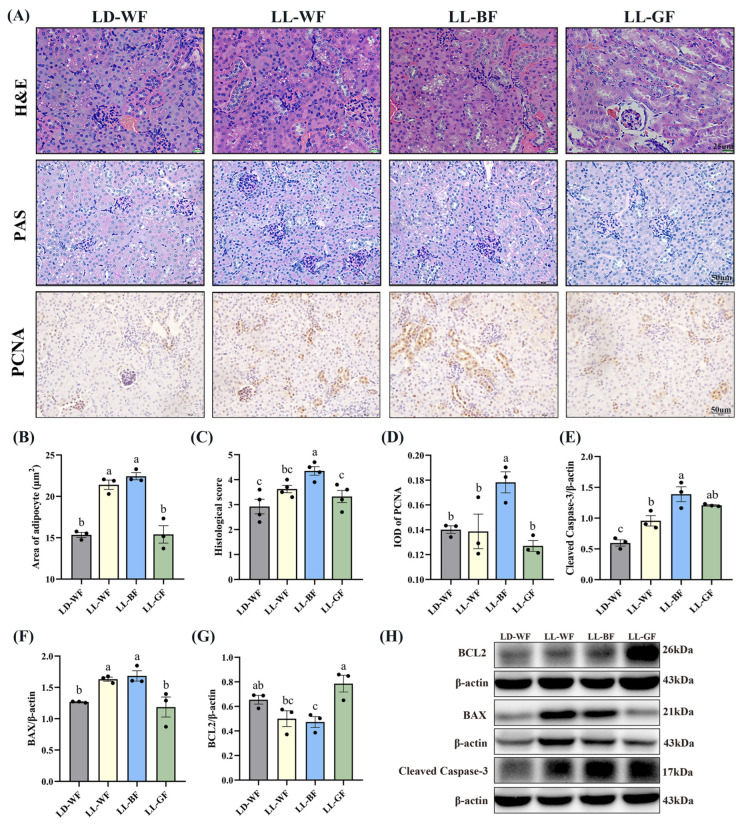
Effects of continuous monochromatic light exposure on renal histology and apoptosis. (**A**) Representative H&E, PAS, and IHC staining of PCNA in the kidney sections (scale: 25 μm in H&E staining; 50 μm in PAS and IHC staining); (**B**) area of adipocyte; (**C**) kidney histological score; (**D**) IOD of PCNA; relative protein levels of the kidney (**E**) Cleaved Caspase-3; (**F**) BAX; (**G**) BCL2; (**H**) Western blot bands of BCL2; BAX; Cleaved Caspase-3. LD-WF: high-fat diet mice under light-dark cycle with white light; LL-WF: high-fat diet mice under continuous white light; LL-BF: high-fat diet mice under continuous monochromatic blue light; LL-GF: high-fat diet mice under continuous monochromatic green light. These results are shown as means ± SEM. Values without the same letter were significantly different (*p* < 0.05) from each other.

Primarily, we observed the histological changes in the kidney. As shown in Figure S2A, H&E and PAS staining demonstrated various degrees of kidney damage and remarkable tubular injury in LD-WF. Compared with LD-WN, LD-WF developed a large area of steatosis in the kidney (Figure S2B,C, *p* < 0.001). As kidney weight gain was observed in the LD-WF group, we focus on cell proliferation in the kidney tissue. Similar results were observed in LD-WF in the integral optical density (IOD) of proliferating cell nuclear antigen (PCNA, Figure S2A,D, *p* < 0.01). To identify the role of different wavelengths of continuous monochromatic light exposure in LD-WF mice, we focus on the development of kidney histological injury. H&E and PAS staining (Figure 2A) indicated that LL-BF showed higher steatosis (Figure 2B,C, *p* < 0.001) and kidney injury histological score than LD-WF. The same trend could be determined in PCNA immunohistochemistry staining in LL-BF (Figure 2A,D, *p* < 0.05).

As the increased kidney histological damage in the LL-BF group was previously observed, we extended the conditions of the experiment we performed in Figure S2 and Figure 2. We used Western blot to test the levels of proteins involved in regulating apoptosis (Figure S2H and Figure 2H). Consistent with what we expected, HFD treatment increased the protein levels of Cleaved Caspase-3 (Figure S2E, *p* < 0.01) and BAX (Figure S2F, *p* < 0.01), which were pro-apoptosis genes, the level of BCL2 was downregulation significantly (Figure S2G, *p* < 0.01). These results shed light on the effects of HFD treatment on the Bcl2/Bax/Cleaved caspase-3 apoptotic signaling pathway. Herein, we explored the effects of persistent light exposure on HFD mice. Remarkably, continuous white light and blue light increased BAX protein levels compared to the LD-WF group (Figure 2F, *p* < 0.05 in LL-WF, *p* < 0.05 in LL-BF). However, the Cleaved Caspase-3 protein level was upregulated, and the BCL2 level was downregulated significantly only in LL-BF mice (Figure 2E,G, *p* < 0.05). Therefore, these results indicated that sustained light exposure could activate pro-apoptotic pathways, and the blue light was even more pronounced than LD-WF.

### 3.3. Continuous Monochromatic Blue Light Increased Renal Fibrosis Induced by HFD

**Figure 3:**
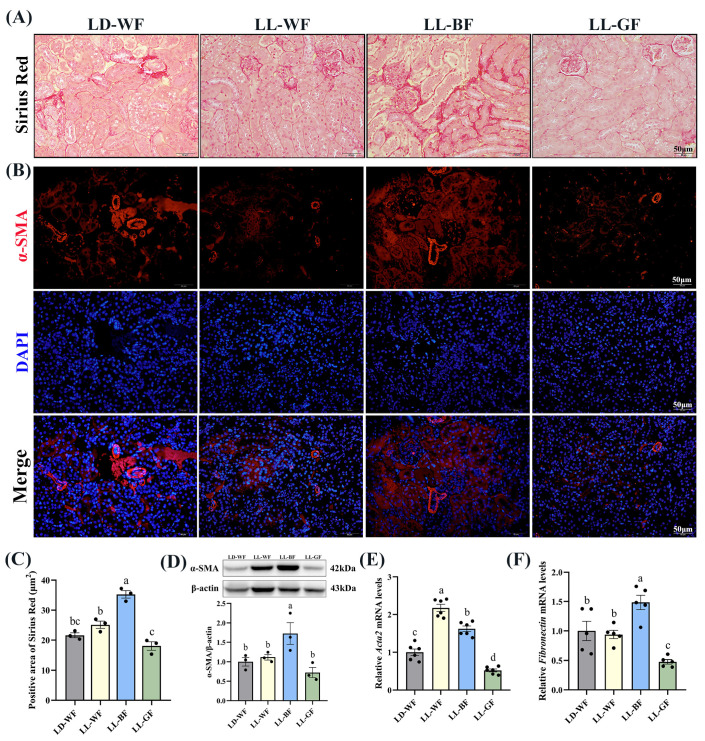
Effects of continuous monochromatic light exposure on renal fibrosis. (**A**) Sirius Red staining in the kidney sections (Scale: 50 μm); (**B**) immunofluorescence of α-SMA in the kidney of mice; (**C**) positive area of Sirius Red staining; (**D**) relative protein levels of α-SMA in the kidney of mice and its Western blot bands (*n* = 3); quantitative relative mRNA levels of (**E**) Acta2 (*n* = 6) and (**F**) Fibronectin (*n* = 5). LD-WF: high-fat diet mice under light-dark cycle with white light; LL-WF: high-fat diet mice under continuous white light; LL-BF: high-fat diet mice under continuous monochromatic blue light; LL-GF: high-fat diet mice under continuous monochromatic green light. These results are shown as means ± SEM. Values without the same letter were significantly different (*p* < 0.05) from each other.

As mentioned above, histological measures revealed more severe renal injuries in LL-BF than in LD-WF. Sirius Red staining revealed increased collagen Ⅰ and Ⅲ deposition in LD-WF mice. In contrast, the kidneys from LD-WF had markedly more staining, particularly around the vasculature (Figure S3A,B, *p* < 0.001). Immunofluorescence labeling showed that a similar trend in α-smooth muscle actin (α-SMA) accumulation was detected in LD-WF (Figure S3C). Moreover, our results showed that LD-WF raised a significantly higher mRNA level of *Acta2* (Figure S3E, *p* < 0.01) and *Fibronectin* (Figure S3F, *p* < 0.05). The protein level of α-SMA has also marginally increased in LD-WF (Figure S3D, *p* < 0.01). As shown in Figure 3A, LL-BF caused an increase in the positive area with Sirius Red staining (Figure 3C, *p* < 0.01) and expression of α-SMA in immunofluorescence colocalization analyses instead of other groups (Figure 3B). Consistent with these notions, the expression of *Acta2* (Figure 3E, *p* < 0.001) and *Fibronectin* (Figure 3F, *p* < 0.01), as well as α-SMA protein level (Figure 3D, *p* < 0.05) were markedly increased in LL-BF. These data demonstrated the establishment of LL-BF-aggravated fibrosis in kidney samples.

### 3.4. Continuous Monochromatic Blue Light Increased Expression of Kidney Injury-Related Genes in the LD-WF Mice

Following up on the findings that histological analysis of the LL-BF mice revealed renal injuries, we performed qRT-PCR. We discovered that the expression of crucial injury response factors, such as kidney injury molecule-1 (*Kim-1*) and lipocalin 2 (*Lcn2*), was higher in the LD-WF group (Figure S4A, *p* < 0.05 and S4B, *p* < 0.001). To comprehensively compare the damage to glomeruli and renal tubules, we observed that the transcripts of essential structural proteins forming the slit diaphragm, such as *Nephrin* (Figure S4C, *p* < 0.05), *Podocin* (Figure S4D, *p* < 0.01), CD2-associated protein (*Cd2ap*, Figure S4E, *p* < 0.01) and *α-Actinin-4* (Figure S4F, *p* < 0.001) were all significantly reduced in LD-WF mice. These results suggested that the kidney structure was damaged in LD-WF. 

**Figure 4:**
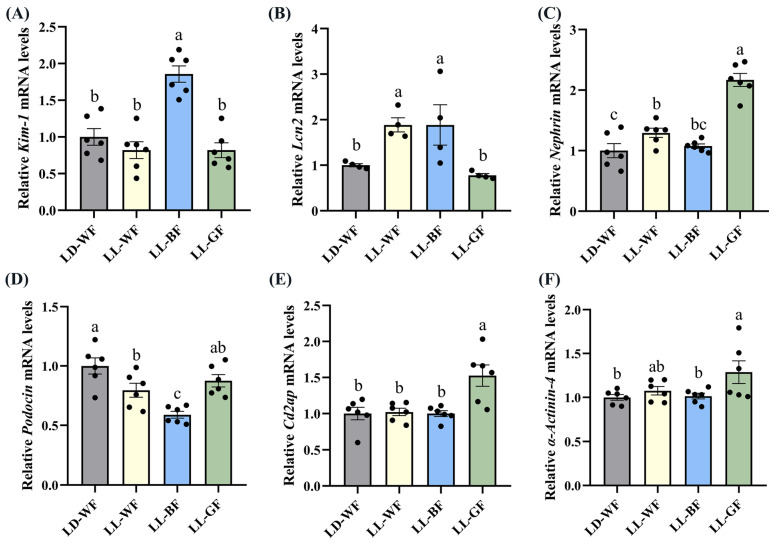
Effects of continuous monochromatic light exposure on expression of kidney injury molecule related genes. Quantitative relative mRNA levels of (**A**) *Kim-1*; (**B**) *Lcn2*; (**C**) *Nephrin*; (**D**) *Podocin*; (**E**) *Cd2ap*; (**F**) *α-Actinin-4*. LD-WF: high-fat diet mice under light-dark cycle with white light; LL-WF: high-fat diet mice under continuous white light; LL-BF: high-fat diet mice under continuous monochromatic blue light; LL-GF: high-fat diet mice under continuous monochromatic green light. These results are shown as means ± SEM. Values without the same letter were significantly different (*p* < 0.05) from each other.

We also found significant differences in the expression of *Kim-1* (Figure 4A, *p* < 0.001), *Lcn2* (Figure 4B, *p* < 0.05), *Podocin* (Figure 4D, *p* < 0.001) between the LD-WF and the LL-BF groups, instead of *Nephrin* (Figure 4C, *p* > 0.05), *Cd2ap* (Figure 4E, *p* > 0.05), and *α-Actinin-4* (Figure 4F, *p* > 0.05). The *p* values refer to the comparisons between the LD-WF and LL-BF groups. Therefore, LL-BF instigates rapid kidney injury, while fewer changes were observed in LL-GF.

### 3.5. Effect of Continuous Monochromatic Blue Light on the Antioxidant Capacity and Inflammation of the Kidney in HFD-Mice

These findings led us to make the following two predictions: persistent blue light upregulates renal injury and may be related to oxidative stress and inflammation; second, it may have some connection with certain hormones and their associated receptors.

To test the first prediction, we examined renal levels of antioxidant parameters. Between LD-WN and LD-WF, the results showed that the antioxidant enzymes, including GSH-Px (Figure S5A, *p* < 0.05), SOD (Figure S5B, *p* < 0.001), CAT (Figure S5C, *p* < 0.001) and T-AOC (Figure S5D, *p* < 0.05) were significantly decreased in LD-WF. However, the MDA content (Figure S5E, *p* < 0.01), which could upregulate oxidative stress levels, increased significantly in the LD-WF group than in LD-WN. We also examined the activation of the NRF2/HO-1 pathway, which was anti-oxidative stress. As shown in Figure S5H, the levels of the NRF2 (Figure S5F, *p* < 0.01) and HO-1 (Figure S5G, *p* < 0.01) protein were down-regulated in the kidney of LD-WF mice. A significant increase in the iNOS protein level (Figure S5H, *p* < 0.01) was observed in the kidney of HFD mice instead of LD-WN. As described above, increasing iNOS expression in the LD-WF group is involved in nitric oxide production and associated with inflammation in the kidney. To investigate whether continuous light affected renal inflammation, we examined the levels of inflammatory cytokines, including pro-inflammatory factors *Il-6* (Figure S5I, *p* < 0.05), *Il-1β* (Figure S5J, *p* < 0.001), *Tnf-α* (Figure S5K, *p* < 0.001) and monocyte chemoattractant protein-1 (*Mcp-1*, Figure S5L, *p* < 0.05) were markedly increased in LD-WF mice; meanwhile, the anti-inflammatory factor *Il-4* was reduced (Figure S5M, *p* < 0.05). Similarly, we found that relative mRNA expression of transforming growth factor β1 (*Tgf-β1*, Figure S5N, *p* < 0.001), crucial for renal fibrosis and inflammation, increased in HFD mice. We then assessed the protein of Toll-like receptor 4 (TLR4, Figure S5O, *p* < 0.05) under these changes brought on by HFD, which regulates obesity-induced inflammation and metabolic dysfunction. The Western blot bands are shown in Figure S5P.

**Figure 5:**
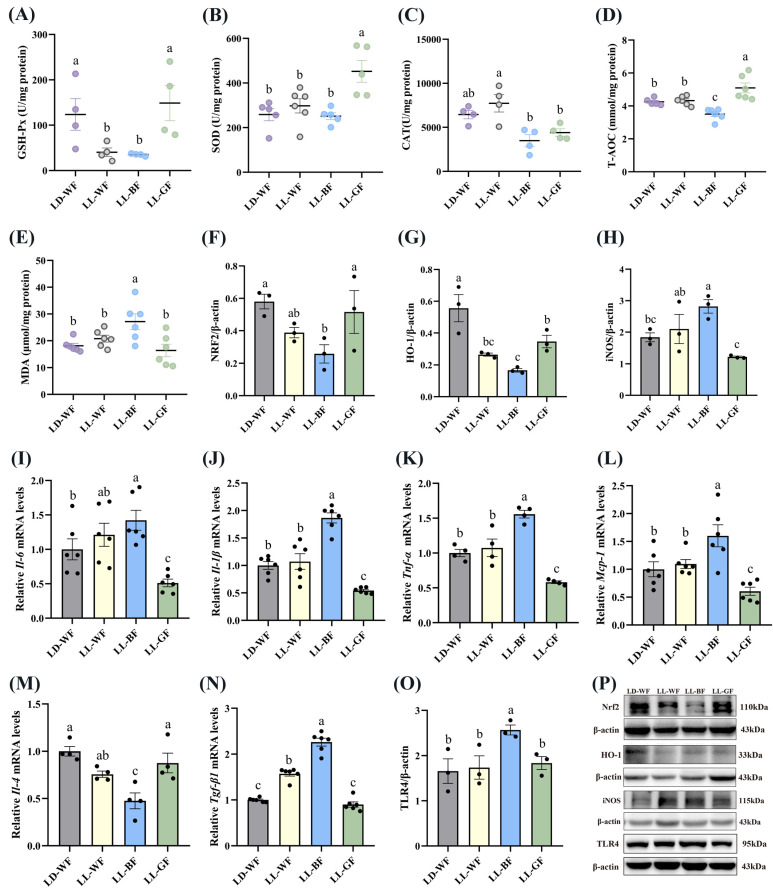
Effects of continuous monochromatic light exposure on oxidative stress and inflammation in the kidney. (**A**) GSH-Px; (**B**) SOD; (**C**) CAT; (**D**) T-AOC and (**E**) MDA levels in the kidney (**F**–**H**) relative protein levels of NRF2, HO-1, and iNOS (*n* = 3), quantitative relative mRNA levels of (**I**) Il-6; (**J**) Il-1β; (**K**) Tnf-α; (**L**) Mcp-1; (**M**) Il-4; (**N**) Tgf-β1 in the kidney; (**O**) relative protein levels of TLR4; (**P**) Western blot bands of NRF2, HO-1, iNOS, and TLR4. LD-WF: high-fat diet mice under light-dark cycle with white light; LL-WF: high-fat diet mice under continuous white light; LL-BF: high-fat diet mice under continuous monochromatic blue light; LL-GF: high-fat diet mice under continuous monochromatic green light. These results are shown as means ± SEM. Differences between the four groups are presented in the form of different letters (*p* < 0.05).

Compared with the LD-WF control group, the GSH-Px (Figure 5A, *p* < 0.05 in LL-WF, *p* < 0.05 in LL-BF) and T-AOC (Figure 5D, *p* < 0.05) expression exhibited marked decreases in LL-BF group. In contrast, SOD (Figure 5B) and CAT (Figure 5C) had no dominant variation tendency. Notably, the MDA concentration in LL-BF was higher than in LD-WF (Figure 5E, *p* < 0.01). Western blot data showed that both NRF2 (Figure 5F, *p* < 0.05) and HO-1 (Figure 5G, *p* < 0.01 in LL-WF, *p* < 0.001 in LL-BF) compared with the LD-WF group were substantially decreased in the kidney of LL-BF mice. The iNOS protein level increased (Figure 5H, *p* < 0.05) in promoting oxidative stress in LL-BF. Accordingly, we observed that blue light could upregulate the *Il-6* (Figure 5I, *p* < 0.05), *Il-1β* (Figure 5J, *p* < 0.001), *Tnf-α* (Figure 5K, *p* < 0.001), and *Mcp-1* (Figure 5L, *p* < 0.01) levels that can promote inflammation compared to the LD-WF group. Surprisingly, these results did not occur in the LL-WF group. In addition, the *Il-4* mRNA level was reduced in LL-BF (Figure 5M, *p* < 0.001). Some other factors, including *Tgf-β1* (Figure 5N, *p* < 0.001) mRNA level and TLR4 (Figure 5O, *p* < 0.05) protein expression, significantly increased with blue light exposure, *p* values refer to the comparisons between LD-WF and LL-BF groups. The Western blot bands are shown in Figure 5P.

Together these observations draw the following conclusions: with the different wavelengths of monochromatic light exposure, we observed that blue light could deteriorate the antioxidant capacity and inflammation in the kidney of HFD mice.

### 3.6. Continuous Monochromatic Blue Light Affected Plasma CORT Concentration and Glucocorticoid Receptors Expression of the Kidney in HFD Mice

**Figure 6:**
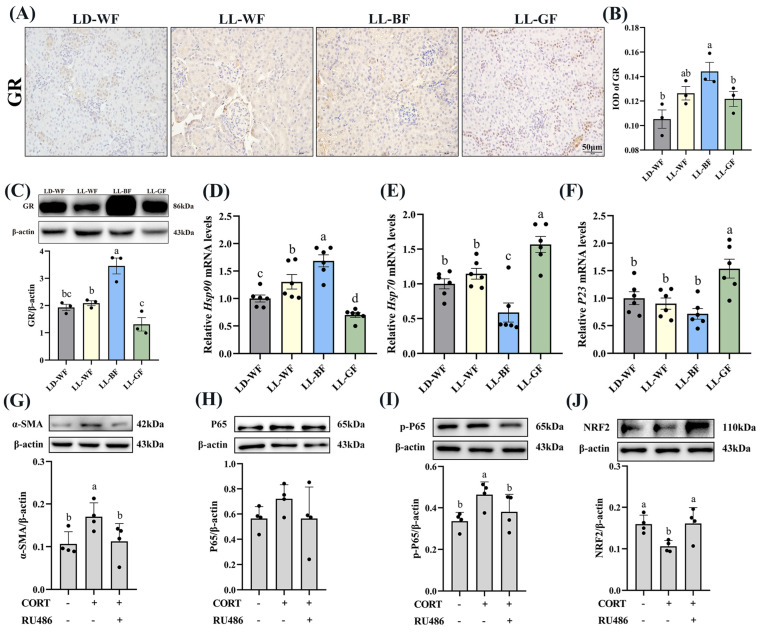
Effects of continuous monochromatic light exposure on expression of corticosterone and glucocorticoid receptors in the kidney and its mechanism. (**A**) IHC staining of GR in the kidney (scale: 50 μm); (**B**) IOD of GR; (**C**) relative protein levels of GR; (**D**–**F**) quantitative relative mRNA levels of Hsp90, Hsp70, and P23. Protein expression levels of α-SMA, P65, p-P65, and NRF2 (**G–J**) were detected by immunoblotting in HK-2 cells (*n* = 4) treated with CORT (10 μM) and RU486 (100 μM). LD-WF: high-fat diet mice under light-dark cycle with white light; LL-WF: high-fat diet mice under continuous white light; LL-BF: high-fat diet mice under continuous monochromatic blue light; LL-GF: high-fat diet mice under continuous monochromatic green light. These results are shown as means ± SEM. Differences between the groups are presented in the form of different letters (*p* < 0.05).

Recent research has shown that glucocorticoids and their receptors are crucial in the pathogenesis of kidney disorders. We measured the plasma corticosterone concentrations to investigate the role of glucocorticoids in HFD mice and the effects of continuous monochromatic light exposure at various wavelengths. As expected, plasma CORT in the HFD-treated group increased significantly compared to the LD-WN mice. Similarly, LL-BF also showed a higher level than LD-WF. Next, we found that IOD in the positive area of glucocorticoid receptors (GR) increased by 78.8% in the LD-WF group compared with LD-WN (Figure S6A,B, *p* < 0.05). The GR protein level was also upregulated in the kidney of HFD mice (Figure S6C, *p* < 0.01). In addition, the relative mRNA levels of heat shock protein 90 (*Hsp90*, Figure S6D, *p* < 0.001) showed an obvious correlation in the LD-WF group, while *Hsp70* (Figure S6E, *p* < 0.001) and *P23* (Figure S6F, *p* < 0.01) showed an opposite trend in expression. To affirm whether the persistent blue light promoted the CORT and its receptors, we examined using IHC analysis. The results indicated that GR expression was more evident in the LL-BF group, not in LL-WF (Figure 6A,B, *p* < 0.05). The GR protein level rose similarly (Figure 6C, *p* < 0.001). *Hsp90* (Figure 6D, *p* < 0.001) mRNA levels significantly increased in LL-BF, while *Hsp70* (Figure 6E, *p* < 0.01) was reversed. There was no significant difference from each other in *P23* in the LL-BF group (Figure 6F, *p* > 0.05).

### 3.7. Corticosterone Induced Oxidative Stress and Inflammation in HK-2 Cells via Glucocorticoid Receptors

Since the LL-BF mice grew up with more CORT in plasma and more oxidative stress and glucocorticoid receptors, they developed chronic inflammation. As we hypothesized, these factors may contribute to kidney damage. To investigate the impact of corticosterone (CORT) and its receptor on renal injury, we exposed HK-2 cells, a renal tubular cell line, to CORT and mifepristone (RU486, an inhibitor of glucocorticoid receptor). α-SMA protein expression was elevated compared to the control (Figure 6G, *p* < 0.05) after being treated with CORT (10 μM) and RU486 (100 μM) and their combination. The expression of inflammation-related proteins was then examined. The level of p-p65 was higher in CORT-treated cells (Figure 6I, *p* < 0.05), while p65 did not vary markedly (Figure 6H, *p* > 0.05). However, the addition of mifepristone reversed this change. We were surprised to discover that the protein levels of NRF2 were lower in the CORT-treated group (Figure 6J, *p* < 0.05). These findings indicated increased that corticosterone levels caused renal fibrosis, inflammation, and oxidative stress via glucocorticoid receptors.

## 4. Discussion

In this research, we examine the impact of constant blue light on the progression of renal disease brought on by HFD. Our data revealed that excessive blue light exposure exacerbated kidney injury and activated pro-apoptotic signaling pathways mainly through elevated plasma corticosterone levels and renal GR expressions, leading to oxidative stress and inflammation. According to our knowledge, this is the first study to establish a link between the change in light wavelength and renal injury in HFD mice under LL conditions.

Many previous studies have reported the adverse effects of a high-fat diet on the kidneys. Sun et al. reported that the HFD diet exacerbated kidney injury by focusing on mitochondrial dysfunction, which has been identified as a major contributor to cell death. As well as the manifestation of kidney injury, including fibrosis, glomerular and tubular lesions, the consequences are the development of Albuminuria [17]. Yamamoto et al. revealed that excessive fat intake induced metabolic diseases through inflammation and cellular damage. Lysosomal disorder and low autophagic flux were significant contributors to the etiology of renal lipotoxicity [22]. The findings suggest that a high-fat diet can lead to kidney injury through various pathways. The most common independent risk factor for chronic kidney disease (CKD) is obesity, which indicates that renal function is negatively impacted by lipotoxicity in the renal parenchyma [22,23]. Compared with LD-WN normal diet control group, our data showed that LD-WF marked increased obesity-related indicators, including body weight, kidney weight, blood glucose, and vacuolar degeneration of kidney tissue sections after 12 weeks of HFD treatment.

However, very few studies have been performed on how monochromatic light affects the kidney. According to Okuliarova et al., prolonged exposure to low light levels at night impaired renal immune and redox homeostasis and deteriorated the biological clock of gradual changes in circulating immune cells [24]. Furthermore, this is the only study we currently know about establishing a link between light and the kidney. Therefore, the function of LL in HFD mouse models was further investigated in our studies. We found that LL-BF upregulated renal function via increasing Scr, Ucr, urinary albumin, and UA. These findings suggested that continuous blue light irradiation led to more severe kidney injury. In addition, the results of the polymerase chain reaction array showed that LL-BF could affect renal morphological changes and structure of glomerulus and renal tubules molecular markers, including *Nephrin*, *Podocin*, *Cd2ap*, and *α-Actinin-4*. Meanwhile, continuous exposure to blue light aggravated renal apoptosis and activated the pro-apoptotic signaling pathway. Similarly, a high-fat diet aggravates renal fibrosis, usually associated with dyslipidemia [25,26]. Consistent with our expectation, LL-BF can aggravate renal fibrosis compared with the LD-WF group. These factors may be critical contributors to CKD [27]. Different wavelengths of light may contribute to developing and promoting obesity among these different external environmental factors. Overall, these data indicated that continuous exposure to blue light promoted the progression of renal dysfunction, which was associated with kidney damage, fibrosis, and apoptosis.

We found that LL-BF could enhance the oxidative stress and inflammation to detect further the mechanisms of persistent blue light on kidney injury. The antioxidation pathway may be necessary for addition to the well-known antioxidant enzymes and oxidation products such as GSH-Px, SOD, CAT, T-AOC, and MDA. The NRF2/HO-1 pathway has been extensively studied in vertebrates [28]. HO-1 is an NRF2-dependent gene that provides cryoprotection and is involved in the progression of oxidative and age-related diseases [29,30]. At the protein levels, our findings revealed that LL-BF also markedly elevated pro-oxidation factor iNOS and decreased the NRF2/HO-1 pathway relative protein expression. Together these observations support the conclusion that excessive blue light exposure-elevated renal injury is at least partly caused by increased oxidative stress.

More attention should be paid to the effect of light on physiological function. Mechanistically, we identified the persistent blue light-exposed kidney and its related cytokines. In brief, LL-BF aggravated inflammation and activated the pro-apoptosis pathway via induction of *Tnf-α*, *Il-6*, *Il-1β*, and *Mcp-1* relative mRNA levels while decreasing *Il-4* levels. Surprisingly, we discovered that *Tgf-β1* mRNA levels were increased. TGF-β1 has been identified as a critical mediator of renal fibrosis and inflammation, primarily inducing fibrosis via its downstream Smad signaling pathway. Overexpression of TGF-β1 developed into progressive renal injury [31]. LL-BF significantly increased the expression of TLR4, which indicated that continuous blue light aggravated the kidney by proposing the inflammatory reaction via activating the TLR4/NF-κB signaling pathway [32]. The current further confirmed that LL-BF aggravated kidney injury by regulating oxidative stress and inflammation.

The light source radiates to the retina and transmits information to the suprachiasmatic nucleus, affecting hormone secretion [33]. Corticosterone is a significant type of hormone essential for the proper functioning of the organism. Previous research and our findings suggest that abnormal light exposure may influence CORT secretion. Glucocorticoids have multiple functions and have been widely used to treat various diseases, including kidney disease [34]. They affect immune cells and induce severe renal injury [35]. However, excessive amounts of glucocorticoids can lead to homeostatic imbalances [36], causing disease processes and organ damage. As we expected, the results suggested that the plasma CORT and GR expression in the kidney of LL-BF mice showed a higher level than the HFD control group. A similar trend was seen in *Hsp90* mRNA expression, and the *Hsp70* trend was the opposite, which co-regulates organ function [37]. The effect of corticosterone on renal tubular epithelial cells was then analyzed. We found that CORT promoted inflammation and fibrosis by upregulating the expression of α-SMA and p-P65. In contrast, the exogenous addition of CORT reduced the expression of NRF2. The above data suggested that CORT affects inflammation and oxidative stress in vitro, maintaining the same trend as the in vivo test results. On the contrary, elevated glucocorticoid receptor levels can dampen antioxidant responses by preventing NRF2-mediated histone acetylation [38]. Besides, increasing glucocorticoid receptors can exacerbate inflammation [39], consistent with our previous results.

Light pollution is an environmental factor that deserves our attention. In our study, sustained blue light could exacerbate HFD-induced kidney damage, which was achieved by promoting oxidative stress, inflammation, activation of pro-apoptotic pathways, and increased secretion of glucocorticoid levels. Our findings fill a gap in prior research on light’s impact on kidneys and guide scientific use of light sources and focus on the dangers of misuse. However, there are still many defects in our study. We found that LL-BF affected the secretion of corticosterone, but the mechanism of the effect is unclear, which is the central task of our future research work.

## 5. Conclusions

Collectively, our study demonstrated that continuous blue light exposure aggravated renal injury and dysfunction by increasing oxidative stress and inflammation, activating the pro-apoptosis signaling pathway in the kidneys of mice that had been given a high-fat diet. Surprisingly, it also increased plasma corticosterone levels and renal glucocorticoid expression. This phenomenon is evidenced by fatty degeneration of renal tissue, decreased mRNA expression of glomerular and tubular-related structural genes, renal fibrosis, and increased renal dysfunction. LL-WF also had a similar result but was less obvious than the blue light. More importantly, our findings have potential value in treating kidney damage due to obesity accompanied by type 2 diabetes. The application of inappropriate ambient lighting has given rise to additional thoughts and filled the paucity of previous studies in this area.

## Figures and Tables

**Table 1:** Sequences of primers used for RT-PCR.

Gene	Product Size	Primer Sequences (5′ to 3′)	Accession No.
*Kim-1*	114	F: ACATATCGTGGAATCACAACGAC R: ACTGCTCTTCTGATAGGTGACA	XM_011248784.3
*Lcn2*	418	F: TGCAGGTGGTACGTTGTGG R: TGTTGTCGTCCTTGAGGC	NM_008491.1
*Nephrin*	213	F: CCCCTCTATGATGAAGTACAAATGGA R: GTACGGATTTCCTCAGGTCTTCT	XM_011250647.3
*Podocin*	232	F: GTGTCCAAAGCCATCCAGTT R: GTCTTTGTGCCTCAGCTTCC	XM_006496684.3
*Cd2ap*	200	F: AGGAATTCAGCCACATCCAC R: TTGAGGGAAACAGTCCCAAC	NM_009847.4
*α-Actinin-4*	189	F: GCCATCCAGGACATCTCTGT R: CCGCAGCTTGTCATACTCAA	XM_006540257.4
*Acta2*	102	F: GTCCCAGACATCAGGGAGTAA R: TCGGATACTTCAGCGTCAGGA	XM_006526606.2
*Fibronectin*	117	F: TCCACAGCCATTCCTGCGCC R: GTTCACCCGCACCCGGTAGC	XM_006495700.4
*Il-6*	200	F: ATAGTCCTTCCTACCCCAATTTCC R: CTGACCACAGTGAGGAATGTCCAC	NM_031168.2
*Il-1β*	116	F: GAAATGCCACCTTTTGACAGTG R: TGGATGCTCTCATCAGGACAG	NM_008361.4
*Tnf-α*	177	F: TCAGCCTCTTCTCATTCCTG R: CAGGCTTGTCACTCGAATTT	NM_013693.3
*Mcp-1*	175	F: CAAGAAGGAATGGGTCCAGA R: TGAGGTGGTTGTGGAAAAGG	NM_011333.3
*Il-4*	102	F: GGTCTCAACCCCCAGCTAGT R: GCCGATGATCTCTCTCAAGTGAT	NC_000077.7
*Tgf-β1*	133	F: CTCCCGTGGCTTCTAGTGC R: GCCTTAGTTTGGACAGGATCTG	XM_036152883.1
*Hsp90*	116	F: TGTTGCGGTACTACACATCTGC R: GTCCTTGGTCTCACCTGTGATA	NM_010480.5
*Hsp70*	219	F: TGGTGCAGTCCGACATGAAG R:GCTGAGAGTCGTTGAAGTAGGC	NM_010479.2
*P23*	120	F: GTTCTTCGGAGAGCACCTGTT R: GAGAGTCCGGTGTCAATCCAG	XM_036154592.1
*Gapdh*	232	F: CCGAGAATGGGAAGCTTGTC R: TTCTCGTGGTTCACACCCATC	XM_036165840.1

F = forward primer; R = reverse primer.

## Data Availability

The data presented in this study are available in this article or Supplementary Material here.

## Supplementary Materials

The following supporting information can be downloaded at: https://www.mdpi.com/article/10.3390/antiox12051018/s1, Figure S1: High-fat diet induces renal dysfunction in the C57BL/6 mice; Figure S2: High-fat diet induces renal histological injury and apoptosis in the C57BL/6 mice; Figure S3: High-fat diet induces renal fibrosis in the C57BL/6 mice; Figure S4: High-fat diet affects expression of kidney injury molecule related genes in the C57BL/6 mice; Figure S5: High-fat diet induces oxidative stress and inflammation in the kidney of the C57BL/6 mice; Figure S6: High-fat diet affects expression of glucocorticoid receptors in the kidney of the C57BL/6 mice.
