# Supplementary material for: Continuous Monochromatic Blue Light Exacerbates High-Fat Diet-Induced Kidney Injury via Corticosterone-Mediated Oxidative Stress
Source: Antioxidants (Basel). 2023 Apr 28;12(5):1018. doi: 10.3390/antiox12051018 (PMC10215342; doi:10.3390/antiox12051018)
Supplement: Supplementary file 1 [file antioxidants-12-01018-s001.zip › antioxidants-2297624-supplementary.pdf]

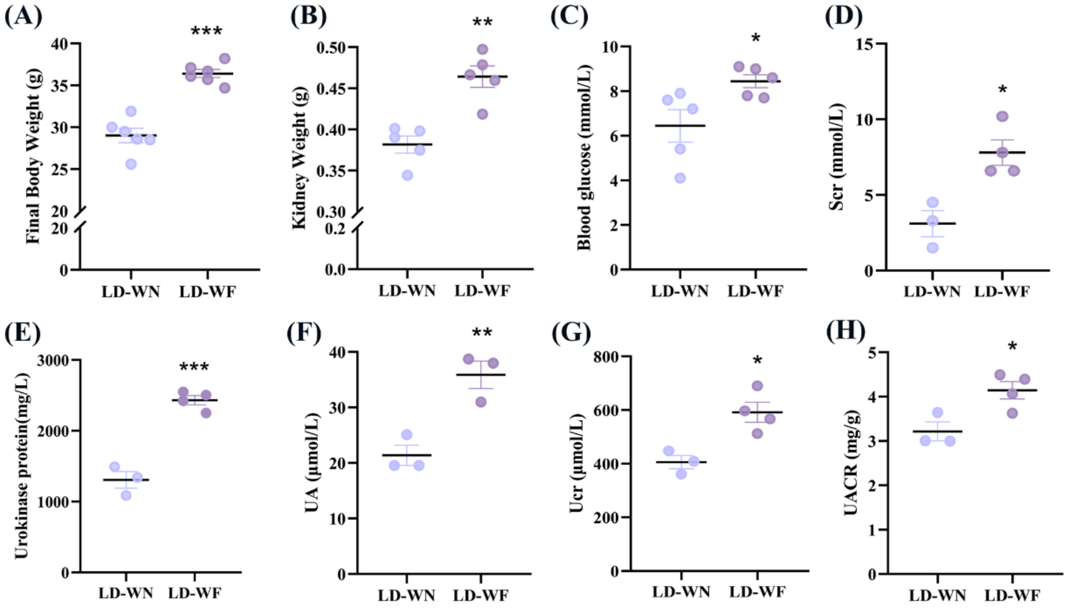

Figure S1. High-fat diet induces renal dysfunction in the C57BL/6 mice. (A) Body weight (n = 6); (B) kidney weight (n = 5) changes at the end of 12 weeks of LD-WF or normal diet (LD-WN) feeding; (C) Changes in fasting blood glucose (n = 5); (D) Scr; (E) Urokinase protein (n = 4); (F) UA (n = 3); (G) Ucr (n = 3); (H) UACR (n = 3) in these mice. LD-WN: normal diet mice under light-dark cycle with white light; LD-WF: high-fat diet mice under light-dark cycle with white light; Scr: serum creatine; UA: uric acid, Ucr: urinary creatine; UACR: urine albumin to creatinine ratio. The results are presented as the means ± SEM. * $p < 0.05$, ** $p < 0.01$, *** $p < 0.001$ versus LD-WN.

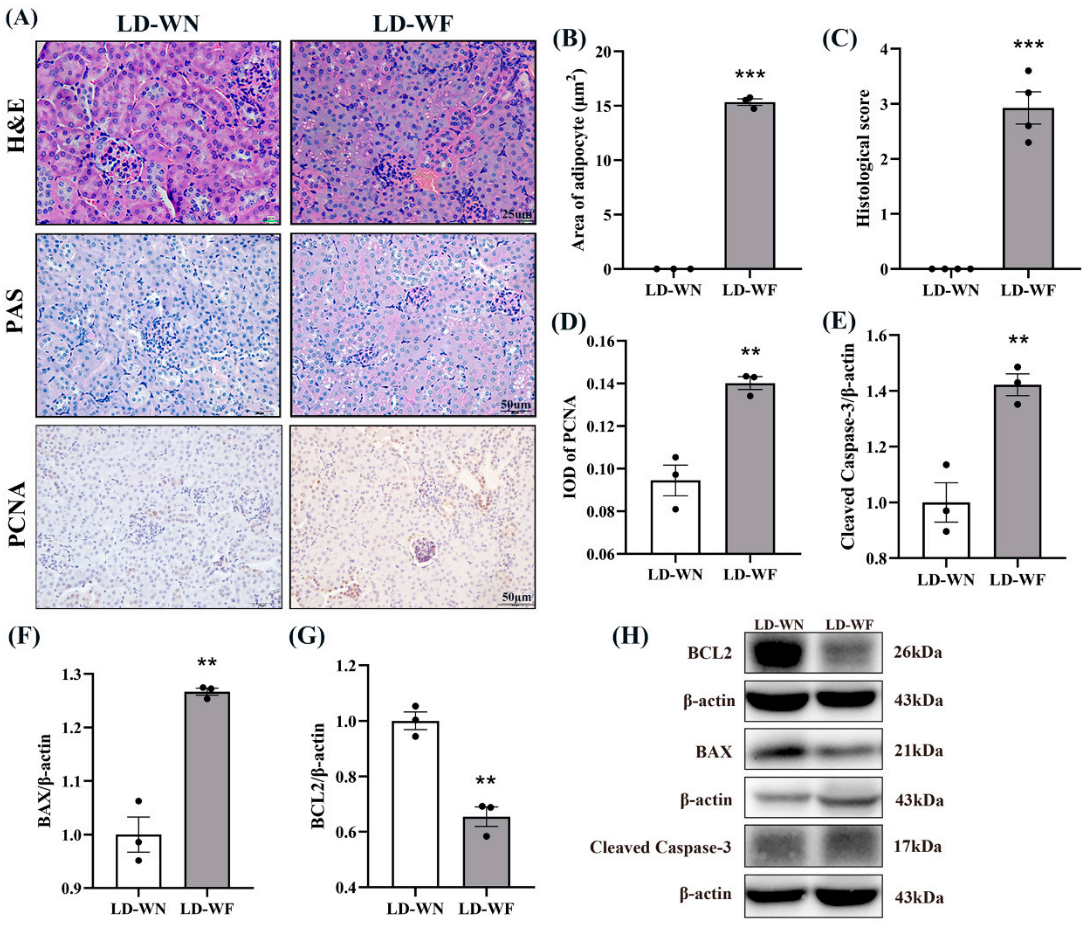

Figure S2. High-fat diet induces renal histological injury and apoptosis in the C57BL/6 mice. (A) Representative H&E, PAS and IHC staining of PCNA in the kidney sections between the LD-WN and LD-WF groups (scale: 25 μm in H&E staining; 50 μm in PAS and IHC staining); (B) area of adipocyte; (C) kidney histological score; (D) IOD of PCNA; relative protein levels of kidney (E) Cleaved Caspase-3; (F) BAX; (G) BCL2; (H) western blot bands of BCL2; BAX; and Cleaved Caspase-3. LD-WN: normal diet mice under light-dark cycle with white light; LD-WF: high-fat diet mice under light-dark cycle with white light. The results are presented as the means ± SEM. * $p < 0.05$, ** $p < 0.01$, *** $p < 0.001$ versus LD-WN.

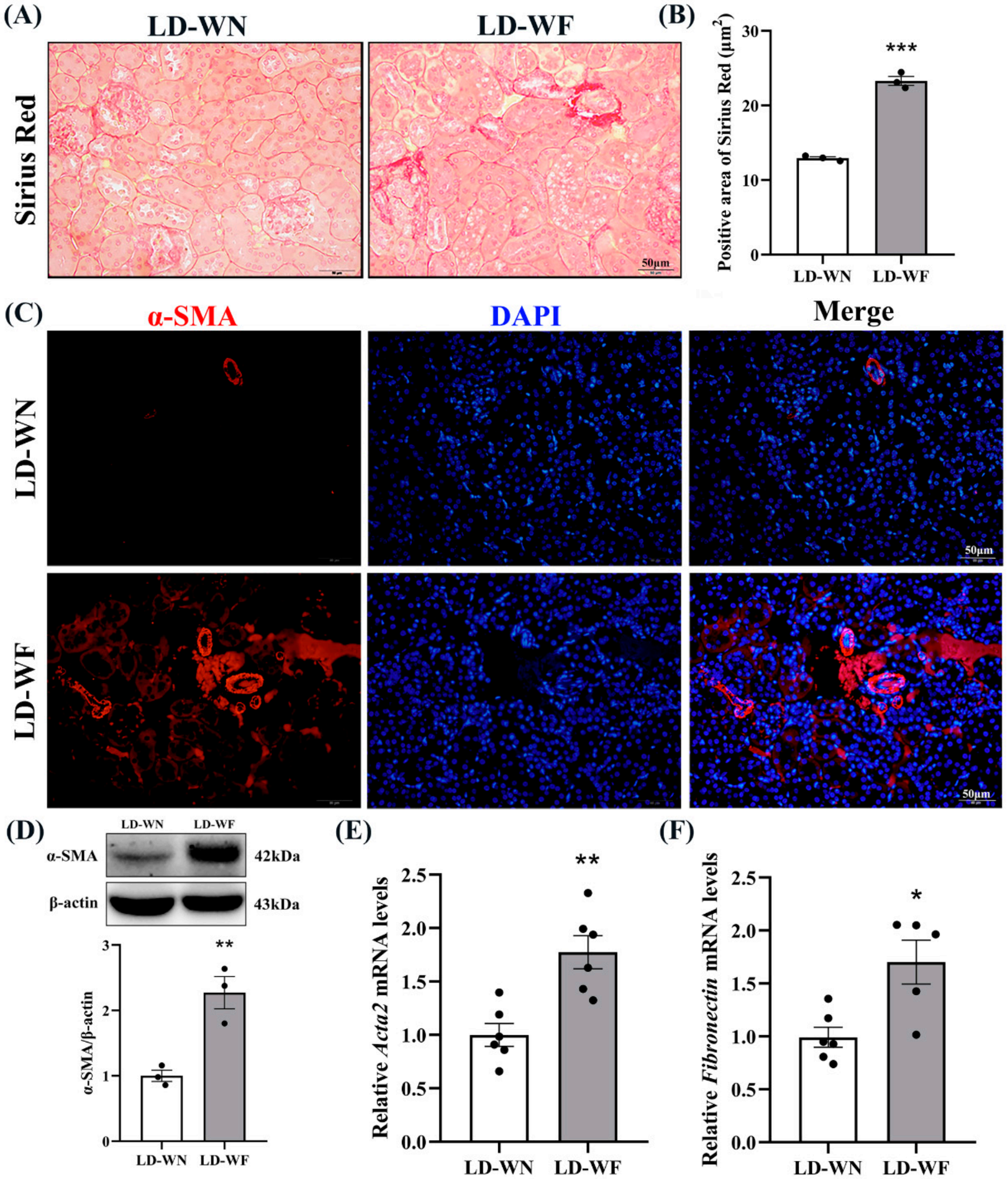

Figure S3. High-fat diet induces renal fibrosis in the C57BL/6 mice. (A) Sirius Red staining in the kidney sections between the LD-WN and LD-WF groups (Scale: 50 μm); (B) positive area of Sirius Red staining; (C) immunofluorescence of α-SMA in the kidney of mice; (D) relative protein levels of α-SMA in the kidney of mice and its western blot bands (n = 3); quantitative relative mRNA levels of (E) *Acta2* and (F) *Fibronectin* (n = 6). LD-WN: normal diet mice under light-dark cycle with white light; LD-WF: high-fat diet mice under light-dark cycle with white light. The results are presented as the means ± SEM. * $p < 0.05$, ** $p < 0.01$, *** $p < 0.001$ versus LD-WN.

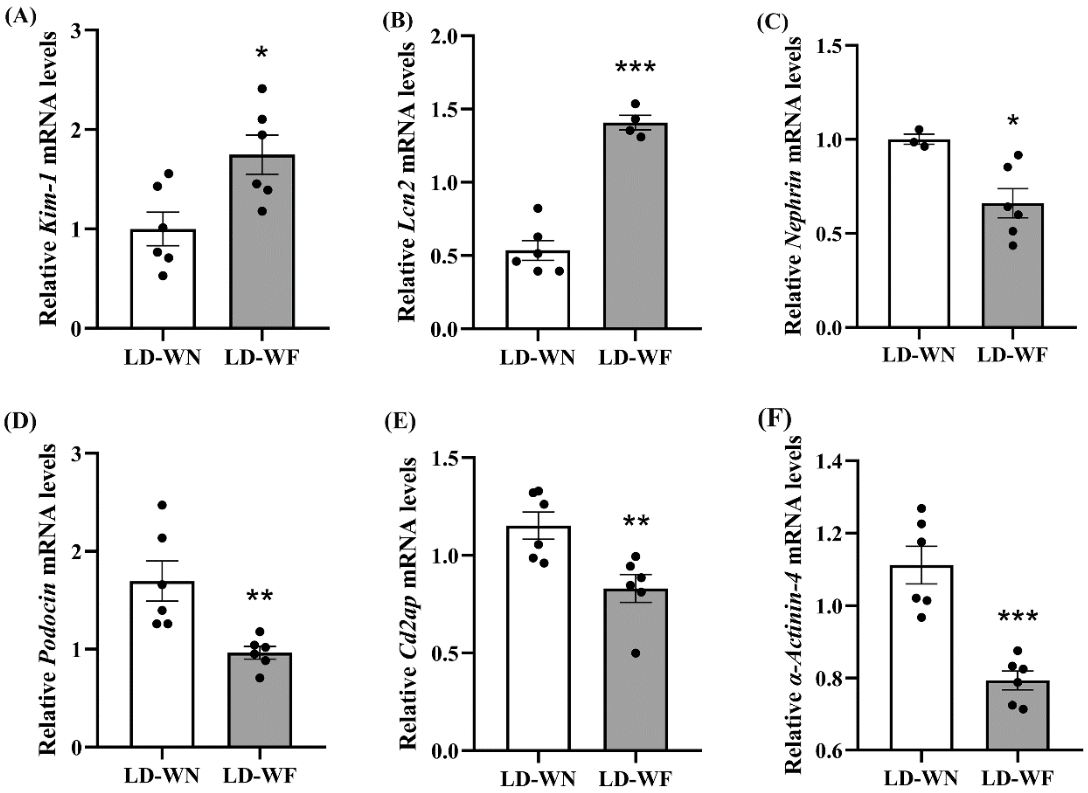

Figure S4. High-fat diet affects expression of kidney injury molecule related genes in the C57BL/6 mice. Quantitative relative mRNA levels of (A) *Kim-1*; (B) *Lcn2*; (C) *Nephrin*; (D) *Podocin*; (E) *Cd2ap*; (F) *α-Actinin-4*. LD-WN: normal diet mice under light-dark cycle with white light; LD-WF: high-fat diet mice under light-dark cycle with white light. The results are presented as the means ± SEM. * $p < 0.05$, ** $p < 0.01$, *** $p < 0.001$ versus LD-WN.

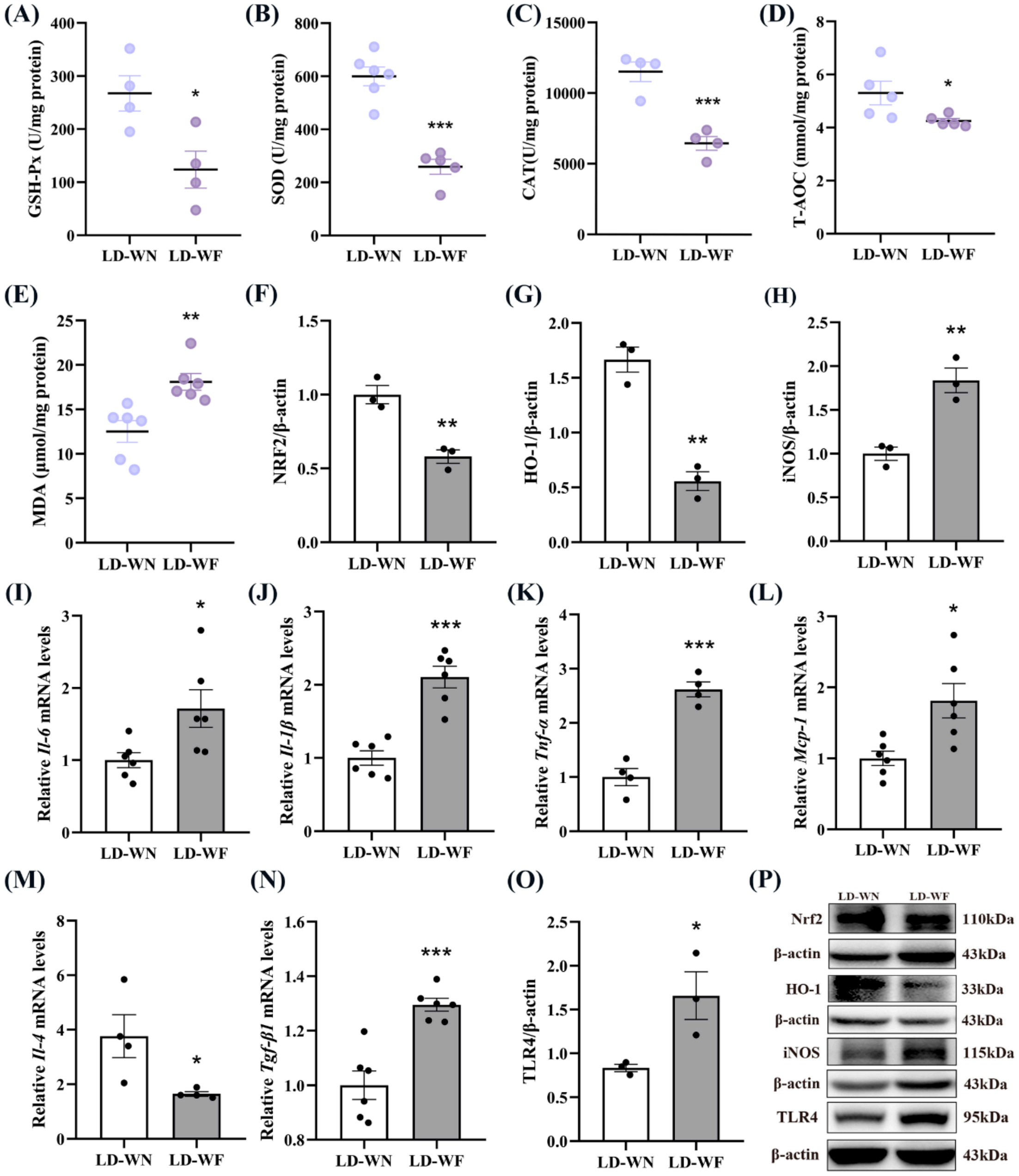

Figure S5. High-fat diet induces oxidative stress and inflammation in the kidney of the C57BL/6 mice. (A) GSH-Px; (B) SOD; (C) CAT; (D) T-AOC and (E) MDA levels in the kidney between the LD-WN and LD-WF groups. (F-H) relative protein levels of NRF2, HO-1 and iNOS (n = 3), quantitative relative mRNA levels of (I) *Il-6*; (J) *Il-1β*; (K) *Tnf-α*; (L) *Mcp-1*; (M) *Il-4*; (N) *Tgf-β1* in the kidney; (O) relative protein levels of TLR4; (P) western blot bands of NRF2, HO-1, iNOS and TLR4. LD-WN: normal diet mice under light-dark cycle with white light; LD-WF: high-fat diet mice under light-dark cycle with white light. The results are presented as the means ± SEM. * $p < 0.05$, ** $p < 0.01$, *** $p < 0.001$ versus LD-WN.

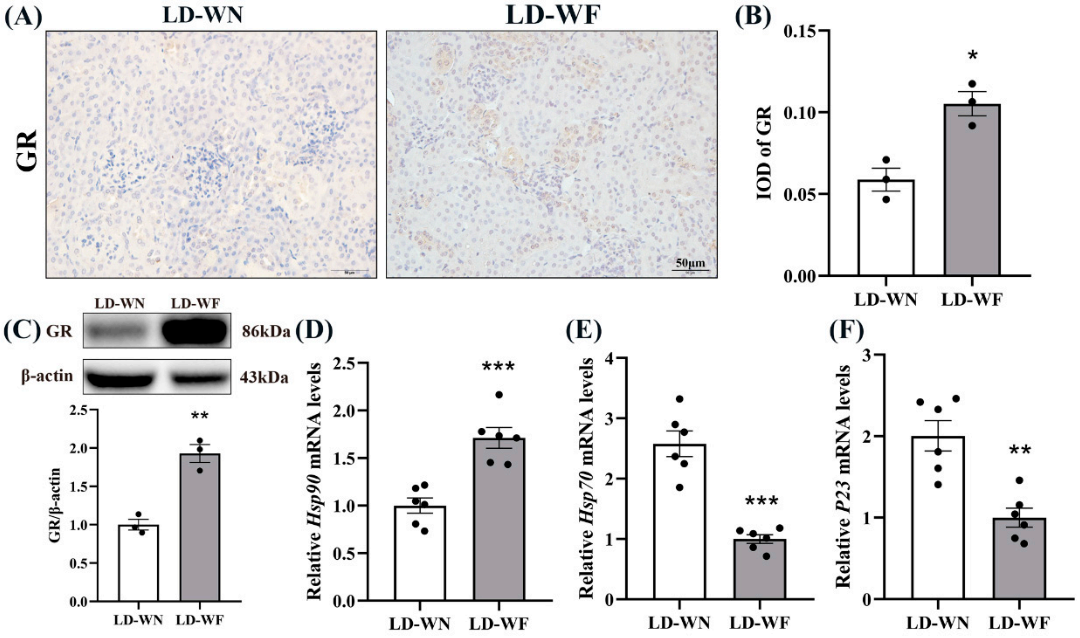

Figure S6. High-fat diet affects expression of glucocorticoid receptors in the kidney of the C57BL/6 mice. (A) IHC staining of GR in the kidney (scale: 50 μm); (B) IOD of GR; (C) relative protein levels of GR; (D-F) quantitative relative mRNA levels of *Hsp90*, *Hsp70* and *P23* in the kidney between the LD-WN and LD-WF groups. LD-WN: normal diet mice under light-dark cycle with white light; LD-WF: high-fat diet mice under light-dark cycle with white light. The results are presented as the means ± SEM. * $p < 0.05$, ** $p < 0.01$, *** $p < 0.001$ versus LD-WN.
