# Supplementary figures and images for: Continuous Monochromatic Blue Light Exacerbates High-Fat Diet-Induced Kidney Injury via Corticosterone-Mediated Oxidative Stress
Source: Antioxidants (Basel). 2023 Apr 28;12(5):1018. doi: 10.3390/antiox12051018 (PMC10215342; doi:10.3390/antiox12051018)

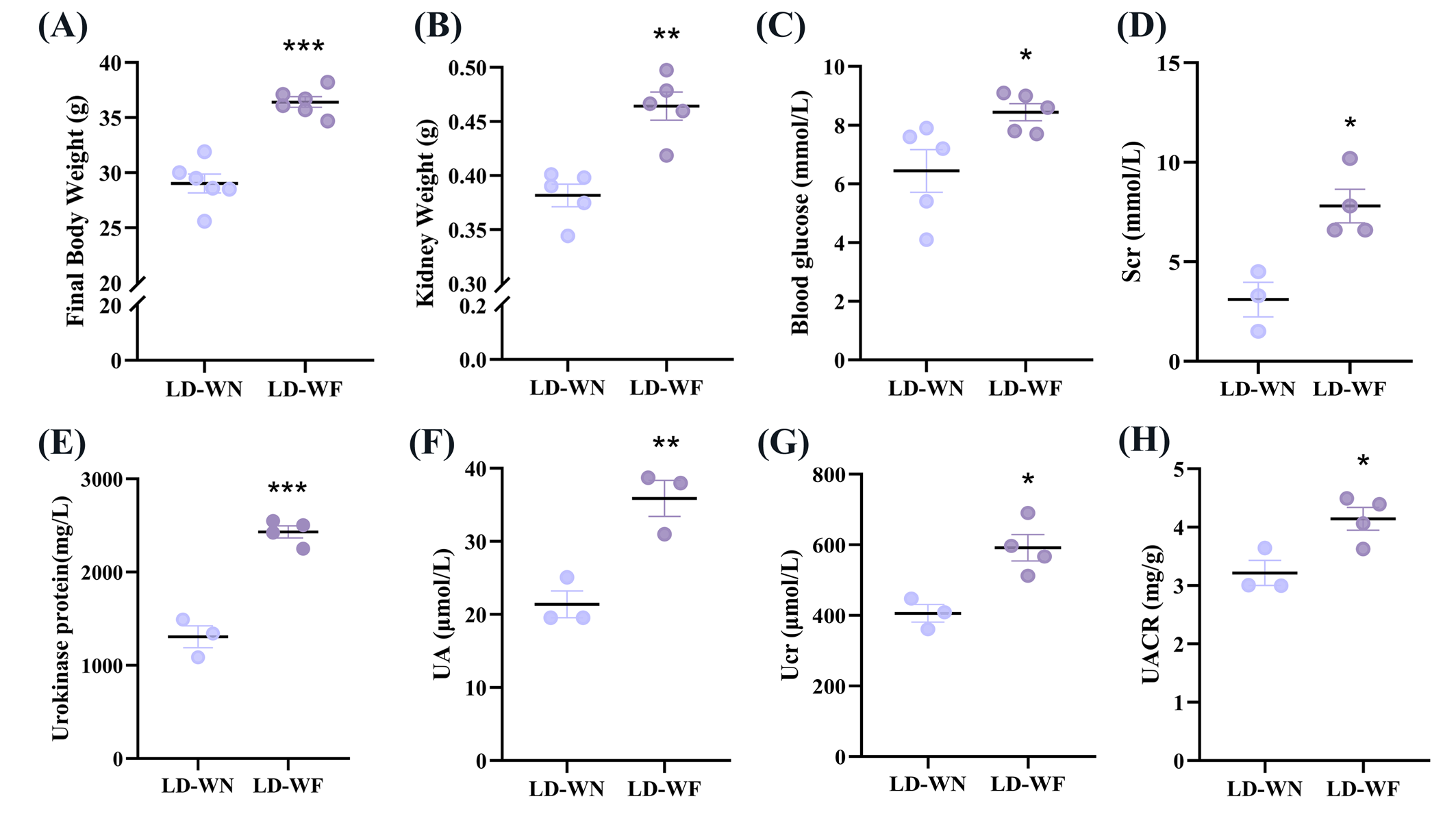

Supplement: Supplementary file 1 [file antioxidants-12-01018-s001.zip › Fig.S1.tif]

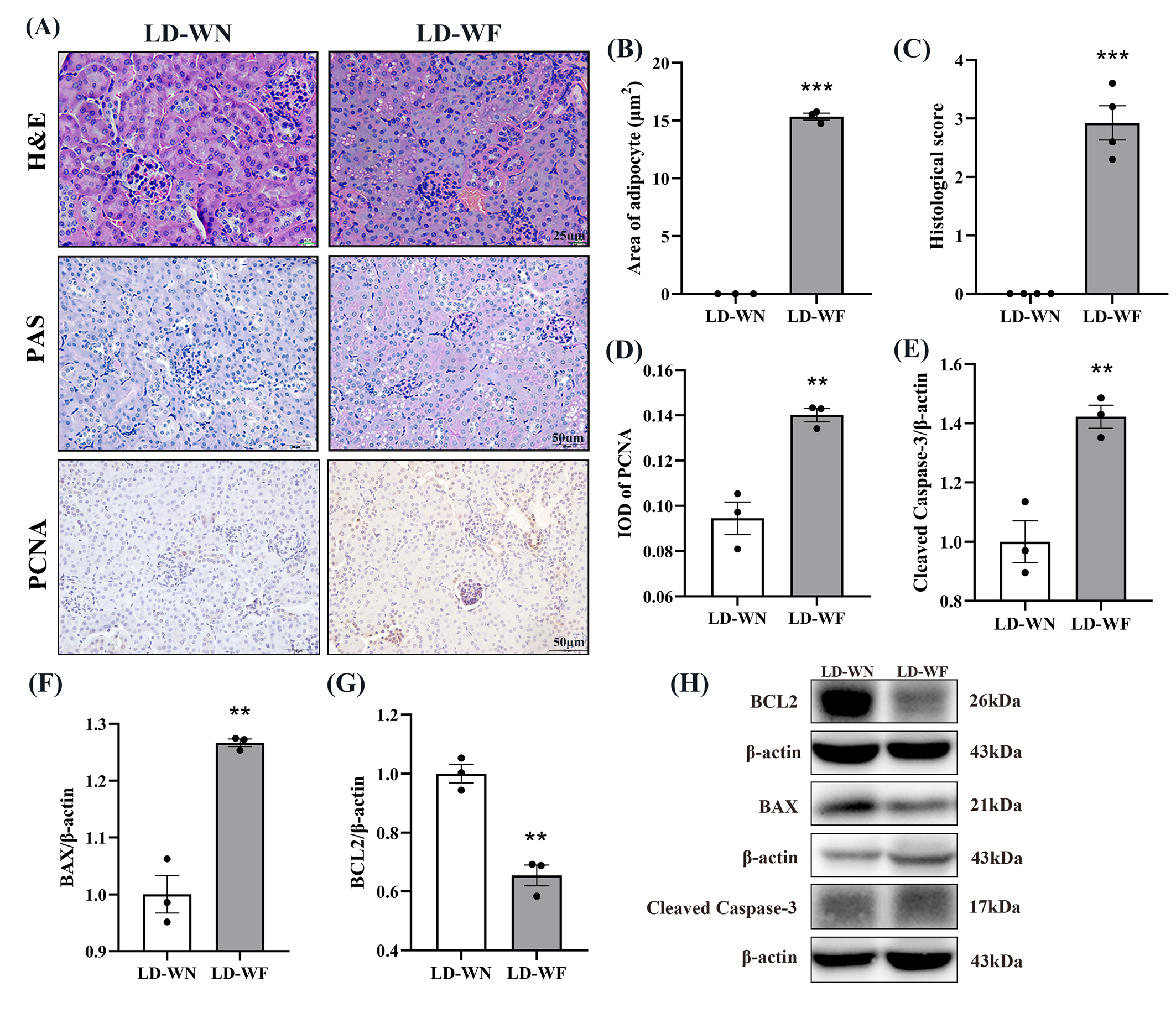

Supplement: Supplementary file 1 [file antioxidants-12-01018-s001.zip › Fig.S2.tif]

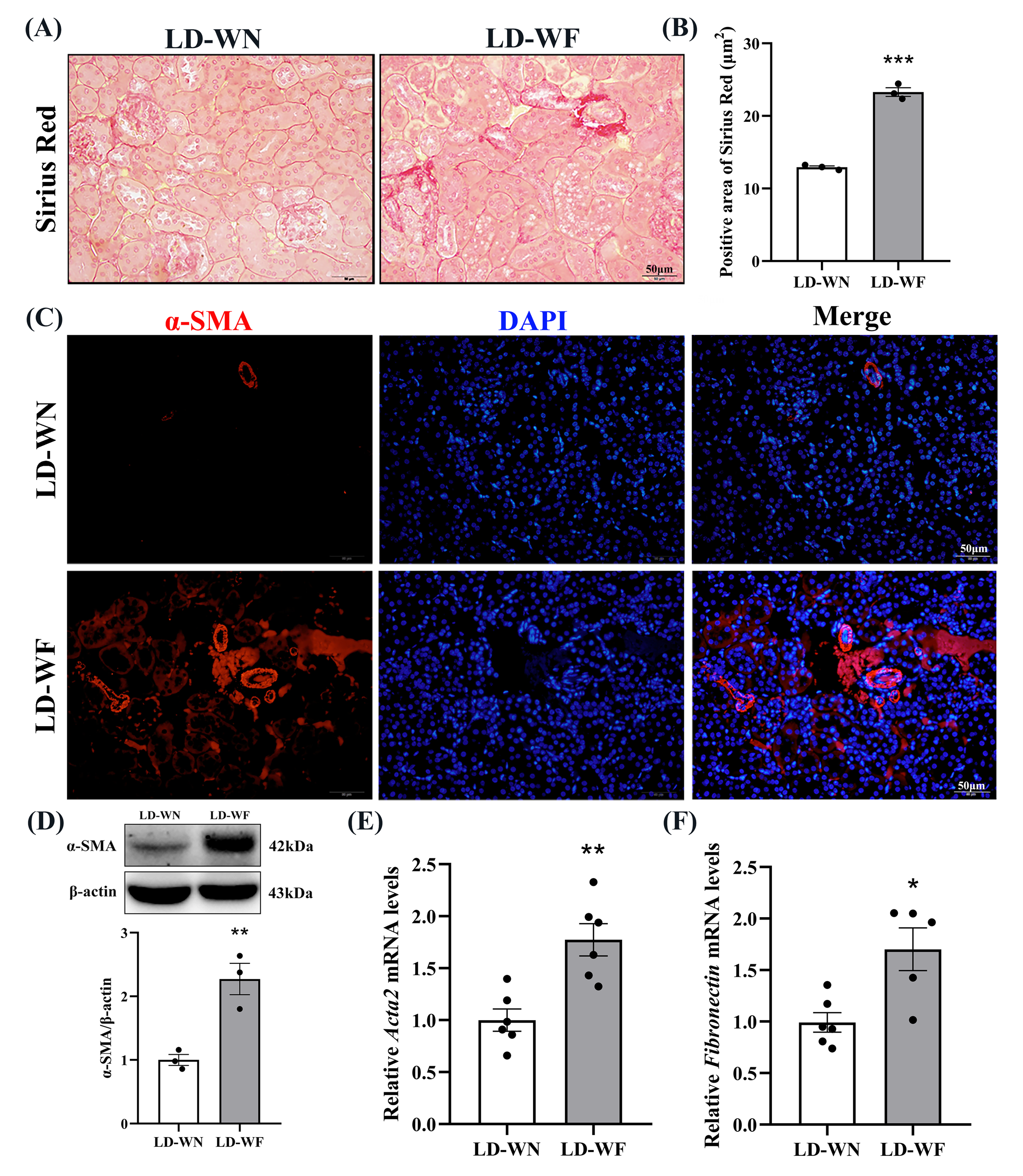

Supplement: Supplementary file 1 [file antioxidants-12-01018-s001.zip › Fig.S3.tif]

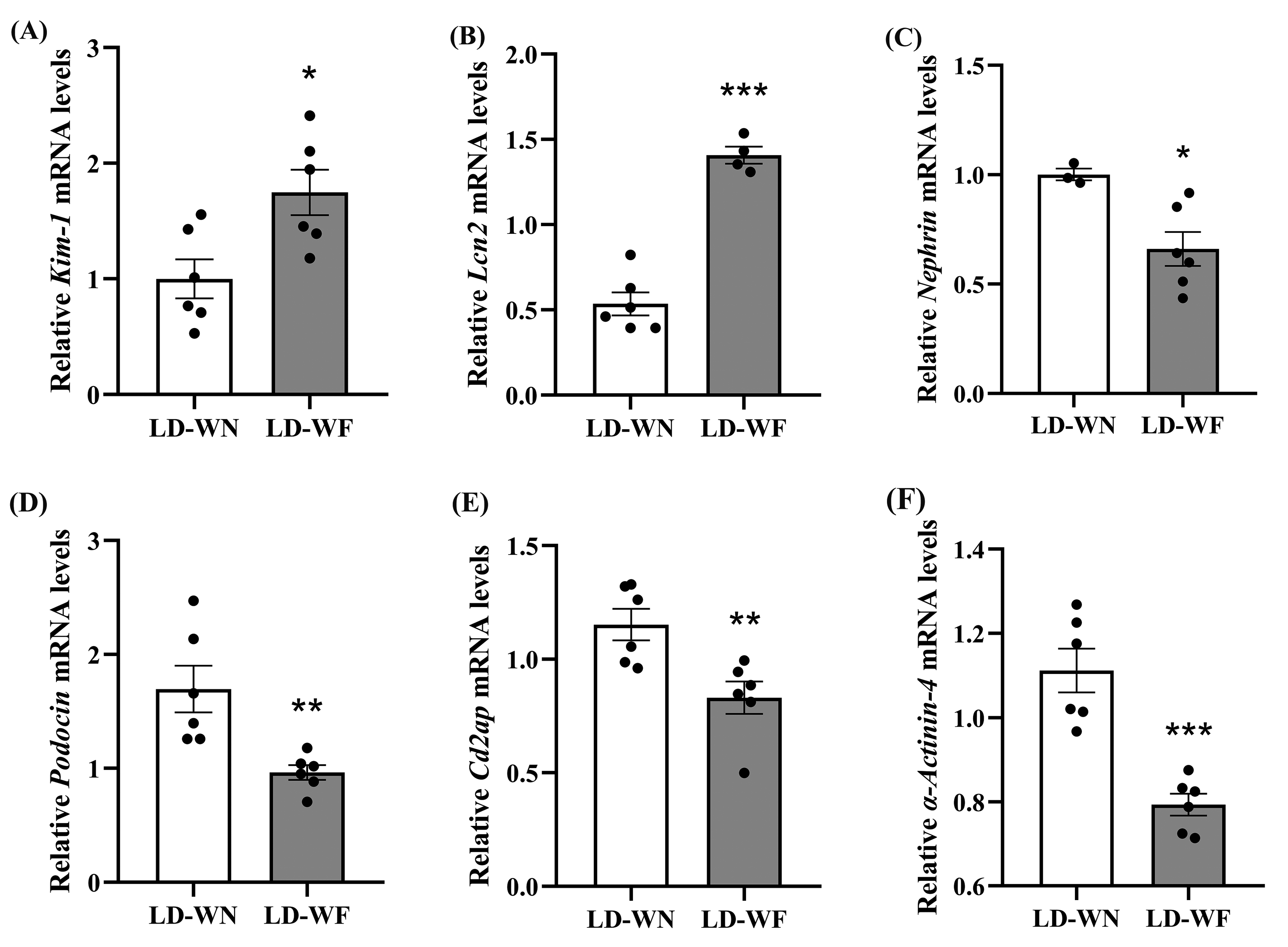

Supplement: Supplementary file 1 [file antioxidants-12-01018-s001.zip › Fig.S4.tif]

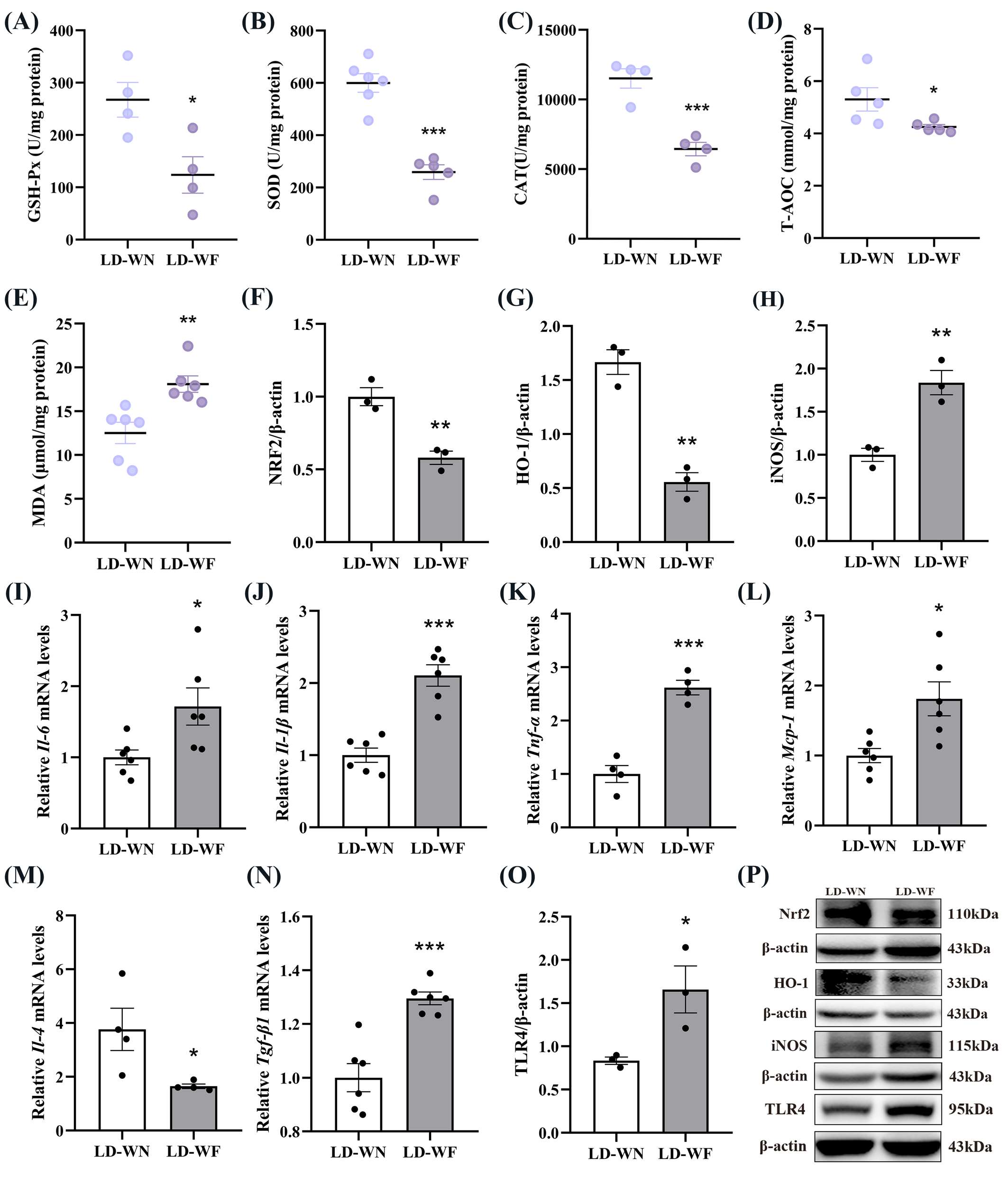

Supplement: Supplementary file 1 [file antioxidants-12-01018-s001.zip › Fig.S5.tif]

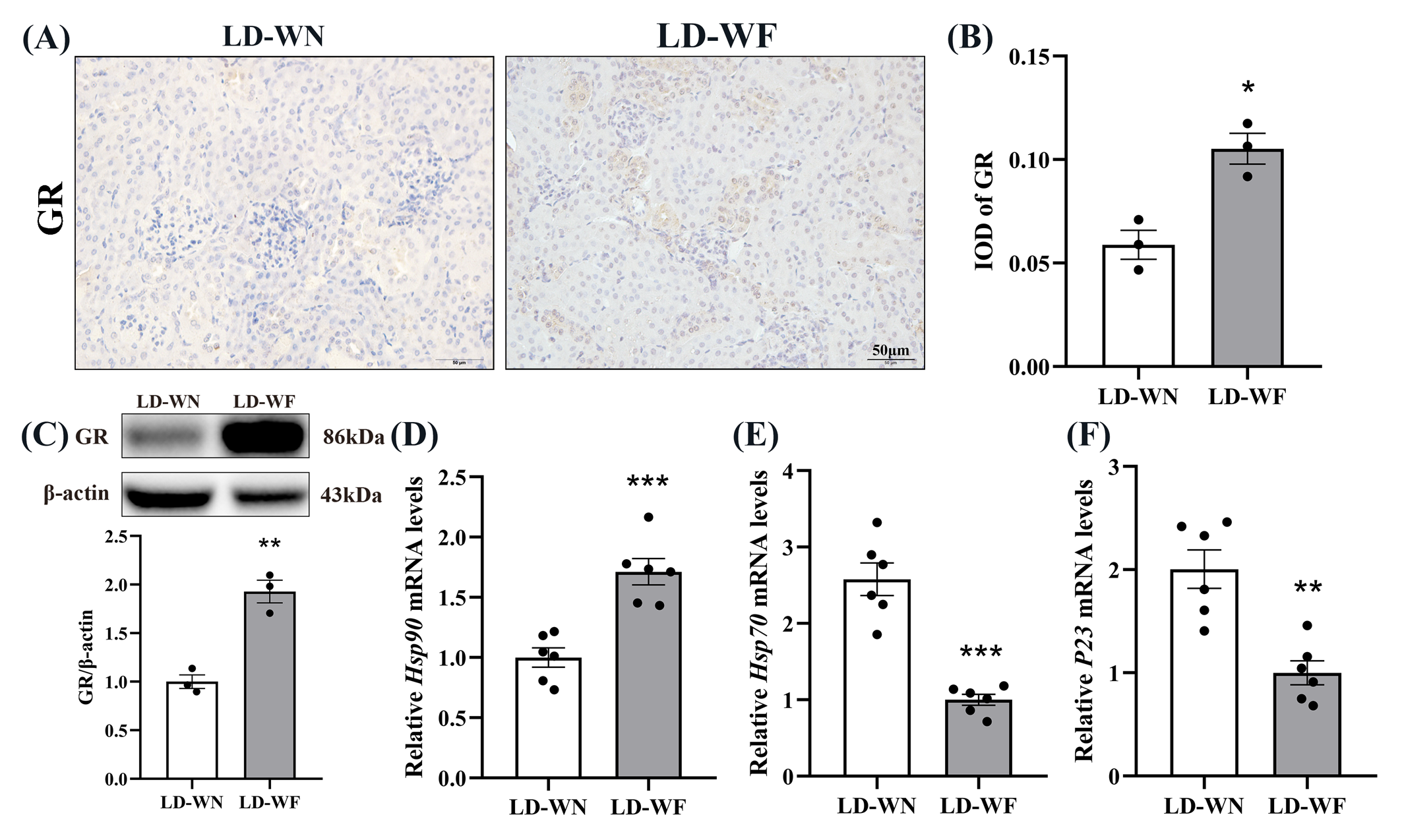

Supplement: Supplementary file 1 [file antioxidants-12-01018-s001.zip › Fig.S6.tif]
